# A biological and genomic comparison of a drug-resistant and a drug-susceptible strain of *Candida auris* isolated from Beijing, China

**DOI:** 10.1080/21505594.2021.1928410

**Published:** 2021-06-01

**Authors:** Shuru Fan, Ping Zhan, Jian Bing, Ning Jiang, Yingnan Huang, Dongke Chen, Tianren Hu, Han Du, Guanghua Huang

**Affiliations:** a Department of Infectious Diseases, Huashan Hospital and State Key Laboratory of Genetic Engineering, School of Life Sciences, Fudan University, Shanghai, China; b Institute of Clinical Medicine and Dermatology Department, Jiangxi Provincial People’s Hospital Affiliated to Nanchang Univercity, Nanchang, China; c Department of Infectious Diseases, Zhongshan Hospital, Fudan University, Shanghai, China; d Department of Laboratory Medicine, Beijing Hospital, National Center of Gerontology, Beijing, China; e Institutes of Biomedical Sciences, Fudan University, Shanghai, China; f Shanghai Key Laboratory of Infectious Diseases and Biosafety Emergency Response, Huashan Hospital, Fudan University

**Keywords:** *Candida auris*, antifungal resistance, morphological phenotype, genomic analysis, clinical isolates

## Abstract

The fungal pathogen *Candida auris* has emerged as a new threat to human health. We previously reported the first isolate of *C. auris* (BJCA001) in China, which belongs to the South Asian clade (I) and was susceptible to all antifungals tested. In this study, we report the isolation of a drug-resistant *C. auris* strain (BJCA002) from the same city (Beijing). Strain BJCA002 belongs to the South African clade (III) and is resistant to fluconazole and amphotericin B based on the tentative MIC breakpoints. Taking advantage of the two isolates with distinct antifungal susceptibility and genetic origins, we performed a biological and genomic comparative study. Besides antifungal susceptibility, strains BJCA001 and BJCA002 showed differences in multiple aspects including morphologies, expression of virulence factors, virulence, mating type, and genomic sequence and organization. Notably, strain BJCA002 was less virulent than BJCA001 in both the *Galleria mellonella* and mouse systemic infection models. Genomic analysis demonstrated that strain BJCA002 but not BJCA001 had multiple mutations in drug resistance-associated genes, including a hot-spot mutation of *ERG11* (VF125AL, namely V125A and F126L) and some missense mutations in *CDR1, MDR1*, and *TAC1*. Notably, strain BJCA001 carried 64 copies of the Zorro3 retrotransposon, whereas BJCA002 had only 3 copies in the genome. Taken together, our findings not only reveal the genetic and phenotypic diversities of the two isolates from Beijing, China, but also shed new light on the genetic basis of the antifungal resistance and virulence of *C. auris.*

## Introduction

*Candida auris* was first reported in Japan in 2009 and is becoming a global threat [1–4]. As of December 2020, *C. auris* cases have been reported in at least 44 countries across six continents based on data from the Centers for Disease Control and Prevention (CDC) (https://www.cdc.gov/). *C. auris* has also caused several outbreaks in hospitals across the globe [5–8]. In 2016 and 2017, the CDC released a couple of clinical alerts to healthcare facilities throughout the United States warning of the emergence of *C. auris* infections.

In addition to its rapid emergence worldwide, the multidrug resistance properties of *C. auris* make it particular problem in clinical settings. Four major genetic clades of *C. auris* have been reported [1,9]. Distinct genetic clades correspond to different geographic regions. Most clinical isolates of *C. auris* are resistant to fluconazole, and some are resistant to two or more classes of antifungal drugs [9,10]. Mutations in the *ERG11* gene (e.g., Y132F, K143R, V125A, and F126L, the V125A and F126L mutations also commonly referred to as VF125AL or F126L), encoding lanosterol demethylase, have been found to be related with fluconazole resistance of *C. auris*. Moreover, these mutations were specifically associated with different genetic clades: V125A and F126L with the South Africa clade (III), Y132F with the South America clade (VI), and Y132F or K143R with the South Asia clade (I) [9,10]. We recently found that *C. auris* could develop fluconazole resistance through an adaptive mechanism of aneuploidy formation [11].

In 2018, we reported the first isolate (BJCA001) of *C. auris* in Beijing, China from the bronchoalveolar lavage fluid (BALF) of a hospitalized woman [12]. Interestingly, strain BJCA001 was found to be susceptible to all tested antifungals used in clinics. In this study, we report a fluconazole-resistant isolate of *C. auris* from a different hospital in Beijing. This strain was isolated from a newborn boy with blood infection. A comparative analysis demonstrated many biological and genomic differences between the newly isolated fluconazole-resistant strain and the previously reported fluconazole-susceptible strain BJCA001.

## Materials and methods

### Strains and culture conditions

*C. auris* strains were routinely grown in YPD medium (20 g/L peptone, 10 g/L yeast extract, 20 g/L glucose; for solid medium, 20 g/L agar was added). The red dye phloxine B (5 mg/L) was added to the solid medium for colony staining. For the morphological assays, approximately 40–80 cells were plated onto each plate (90 mm in diameter) and incubated at 25°C for seven days.

To evaluate the cell size of *C. auris*, cells were grown to the stationary phase in liquid YPD or YCB-BSA medium at 37°C and then inoculated into liquid YPD or YCB-BSA medium for 24 hours of growth at 25°C and 37°C. Both the cell width and length were examined.

## Whole-genome sequencing

Yeast-from cells of *C. auris* were grown on YPD medium at 37°C for 16 hours. Genomic DNA was extracted using the TIANamp Yeast DNA Kit (TianGen Biotech, Beijing, China) according to the company’s protocol. Briefly, yeast-form cells (collected from 10 mL culture) was resuspended, treated with the lysis buffer, and incubated at 37°C for 40 min. The samples were gently mixed by brief vortexing and cell debris was spun down by centrifugation. The supernatant was transferred to the DNA binding column for centrifugation. Genomic DNA was then released with TE buffer. For genome sequencing, a paired-end library with an average insert size of 300 bp was prepared and sequenced using the Illumina NovaSeq platform. In addition, single-molecule real-time (SMRT) sequencing was performed by Beijing Novogene Bioinformatics Technology Co., Ltd. (Beijing, China) using the PacBio Biosciences (PacBio) Sequel small-molecule real-time sequencing system (Pacific Biosciences, Menlo Park, CA, USA). The sequence data generated from the Illumina platform were used to proofread the PacBio assembly sequence.

## Genomic assembly

The PacBio sequence data were error-corrected, trimmed, assembled and scaffolded using Canu v1.8 software guided by a genome size of 12.5 Mb [13]. Illumina PE reads were quality trimmed for random hexamer primers on the 5′ read end using Trimmomatic v0.22 [14]. The high-quality reads were aligned to the Canu genome assembly using Bwa v0.7.17 [15] and filtered for concordant PE read alignments using SAMTools v1.8 [16]. The Canu genome assembly was additionally corrected with the high-quality Illumina alignments using Pilon v1.23 [17] to generate a final polished sequence assembly with SNP and INDEL corrections.

## Gene prediction

Based on the final assemblies, proteins and coding genes were predicted using the program AUGUSTUS v3.2.1 [18]. Putative ORFs were searched against the NR database of NCBI (http://www.ftp.ncbi.nlm.nih.gov/blast/executable s/blast +/LATES T/) and *Candida* genome database (http://www.candidagenome.org/).

## Whole genome synteny and divergence analysis

The final assemblies of strains BJCA001 and BJCA002 were aligned with the open-source MAUVE aligner version 2.3.1 using a progressive algorithm [19]. The alignment was performed using a skip-refinement and seed-weight of 15 to avoid spurious alignments while other mandatory arguments were run on the program default.

To calculate the genomic divergence of different regions between the two isolates, we broke the assembly of BJCA001 into 5-kbp sliding windows with 1000-bp step. The SNPs of each window compared with the BJCA002 assembly were extracted with the open source software package MUMmer, version 4.4 [20]. The average divergence for each chromosome and the whole genome were calculated based on the SNP density.

The duplication-removed BAM datasets were used to estimate the copy number variation (CNV) for each isolate. Genomic regions with CNV were identified with the Splint script to avoid the “smiley pattern” bias [21]. The read depth by 1000-bp nonoverlapping windows across the genome was generated with default internal parameters.

## Reference-based alignment, variant calling, and Phylogenetic analysis

A total of 24 samples (including BJCA001 and BJCA002) representing the five major clades of *C. auris* were analyzed. The DNA sequence information was retrieved from the NCBI SRA database and previous publications [9,22]. Raw reads were trimmed to remove low-quality (Phred score ≤10), ambiguous, and adaptor bases using the FASTX-Toolkit v0.0.14 (http://hannonlab.cshl.edu/fastx_toolkit/index.html). The clean reads obtained were mapped to the CDC_B8441 genome (https://www.ncbi.nlm.nih.gov/assembly/GCA_002759435.2/) using Bwa v0.7.17 [15] with default settings. SAMTools v1.8 [16] was employed to convert the alignment results into BAM format and Picard Tools v1.56 (http://picard.source-forge.net) was used to remove duplicated sequences. The SAMTools and Genome Analysis Toolkit (GATK v2.7.2) program [23] were used to detect the SNPs and INDELs. For GATK, HaplotypeCaller was used and the ploidy was set as “1”. The parameters “stand_call_conf” (thresholds for low – and high-quality variation loci) and stand_emit_conf (minimum Phred-scaled confidence threshold) were set to 50.0 and 20.0, respectively. The variation sites with a coverage depth ≥20 were retained for the subsequent analyses and final extraction.

Phylogenetic trees were constructed based on the whole genome SNPs of the samples. RAxML v8.1.6 (1000 bootstrap replicates) with the GTR model of nucleotide substitution and γ-distributed rates among sites was used for the analysis [24].

## Minimal inhibitory concentration (MIC) assay

MIC assays were performed according to the CCLS document M27-A3 with slight modifications [11,25]. Briefly, *C. auris* cells of each strain were initially patched on nutrient agar plates at 35°C for 24 hours. Approximately 500 cells were inoculated into 0.2 mL RPMI 1640 medium (w/v, 1.04% RPMI 1640, 3.45% MOPs, using NaOH for pH adjustment to 7.0) in a 96-well plate for MIC testing. Different antifungals in a series of concentrations (from 0.016 to 128 μg/mL) were used. Cells were incubated at 35°C in air for 24 hours. *Candida krusei* ATCC 6258 and *Candida parapsilosis* ATCC 22,019 served as quality controls.

## Secreted aspartyl proteinase (SAP) activity assay

SAP activity was determined using the YCB-BSA method as described previously [26]. Briefly, cells of *C. auris* were initially grown on YPD medium at 30°C for 48 hours. Approximately 5 × 10^6^ cells of each strain in 5 μL ddH_2_O were spotted onto YCB-BSA medium plates and cultured at 25°C, 30°C, or 37°C for seven days. The width of the BSA precipitation rings (halos), which reflect the level of SAP activity, was measured. Three biological repeats were evaluated.

## *Galleria mellonella* infection model

*Galleria mellonella* in the final instar larval stage were purchased from Tianjin Huiyu biological technology Co. LTD. (Tianjin, China). Larvae with a similar size (0.3 g-0.4 g) were used for infection assays. *C. auris* cells were incubated at 30°C with shaking to an OD_600_ = 1.8. *C. auris* cells (1 x 10^6^ in 10 µL 1 x PBS) were washed twice with 1 x PBS and injected into each larva. After injection, the larvae were placed in plastic culture dishes and incubated at 37°C or 25°C in the dark. The survival rates were determined based on the daily records.

## Mouse systemic infection

All animal experiments were performed according to the guidelines approved by the Animal Care and Use Committee of the School of Life Sciences, Fudan University. Mouse systemic infection experiments were performed as described in our previous report [12]. Briefly, five BALB/c female mice (5 weeks old, 13–16 g) were used for intravenous infection of each *C. auris* sample. Each mouse was injected with approximately 2 × 10^7^ fungal cells in 250 μL 1 x PBS via the lateral tail vein. The mice were humanely killed at 24 hours post-infection. The brain, kidney, spleen, liver, and lung of each infected mouse were removed, weighed, and homogenized for determination of colony-forming unit (CFU) on YPD medium.

## Results

### *Isolation of the fluconazole-resistant strain BJCA002 of* C. auris *in Beijing, China*

A male infant was born premature at 33 weeks (gestational age) by natural labor at Beijing Hospital. He was admitted to the neonatal intensive care unit (NICU) and monitored for vital signs. Meropenem and vancomycin were prescribed to prevent bacterial infection. On day 2 after birth, fluconazole was used to prevent fungal infection with intermittent usage of 12 mg per 72 hours. After 12 days of admission, yeast species were isolated from blood cultures, although the β-D-glucan test was negative. The isolates were identified as *C. auris* by the MALDI-TOF MS platform (4.15 version, Bruker, Germany) and verified by molecular identification methods. The sequences of the internal transcribed spacers (ITS) showed 100% identity to some reported *C. auris* isolates [9,27]. This isolate was referred to as BJCA002.

**Figure 1.**
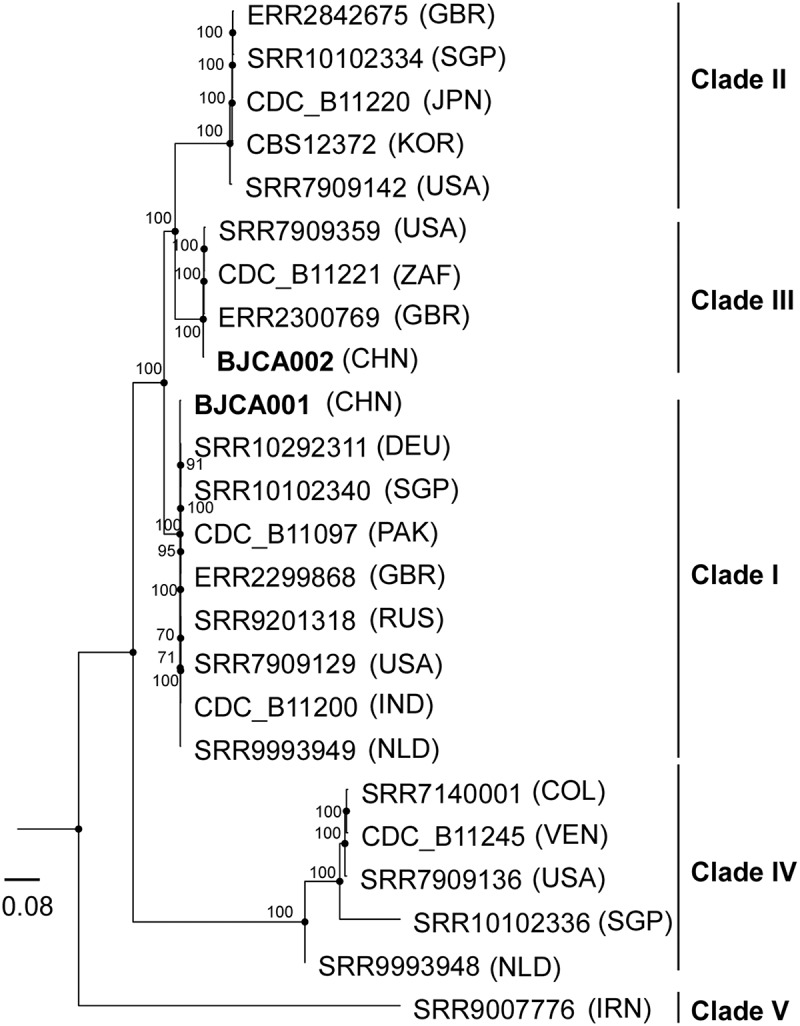
**Phylogenic analysis of two strains isolated from Beijing, China and representative isolates of major clades of *C. auris***. The two strains (BJCA001 and BJCA002) isolated from Beijing are highlighted in bold. The phylogenic tree was generated using the program RAxML v7.3.2 with 381,150 SNPs. The generalized time-reversible (GTR) model, a Gamma distribution, and 1000 bootstraps were used to construct the support of the phylogenetic relationships. Countries from which the isolates originated are indicated in brackets. CHN, China; USA, United States of America; GBR, United Kingdom; JPN, Japan; KOR, Korea (South); SGP, Singapore; ZAF, South Africa; DEU, Germany; PAK, Pakistan; IND, India; RUS, Russia; NLD, Netherlands; IRN, Iran; COL, Colombia; VEN, Venezuela. The scale bar represents the expected number of substitutions per nucleotide position

Phylogeny analysis demonstrated that strain BJCA002 belonged to the South African clade (III, Figure 1), whereas the isolate from Beijing, China previously reported by our group belongs to the South Asian clade (I). Therefore, there are at least two distinct genetic clades of *C. auris* in Beijing. We then tested the antifungal susceptibility of BJCA002 to nine different drugs (Table 1). The minimum inhibitory concentrations (MICs) of fluconazole and amphotericin B of strain BJCA002 were 64 mg/L and 4 mg/L, respectively. These values were higher than the MIC breakpoints determined for other *Candida* species, implying that the strain is multidrug-resistant. The MICs of itraconazole, posaconazole, voriconazole, 5-flucytosine, caspofungin, micafungin, and anidulafungin of strain BJCA002 were equal to or less than 2 mg/L (Table 1). Taken together, compared with strain BJCA001, BJCA002 had relatively higher MICs for all the tested drugs, suggesting a reduced susceptibility to antifungals.

**Table 1.** Antifungal susceptibility testing of *C. auris* strains BJCA001 and BJCA002

	FLC	ITC	POS	VRC	AMB	5-FC	CAS	MFG	AFG
**BJCA002**	64	2	1	0.5	4	0.25	1	0.5	0.5
**BJCA001**	2	0.03	0.02	0.02	0.25	<0.06	0.06	0.06	0.12

MICs (minimal inhibitory concentrations, mg/L) are shown. FLC, fluconazole; ITC, itraconazole; POS, posaconazole; VRC, voriconazole; AMB, amphotericin B; 5-FC, 5-flucytosine; CAS, caspofungin; MFG, micafungin; AFG, anidulafungin.

## Comparison of the morphologies of strains BJCA002 and BJCA001

Given the differences in the genetic background and antifungal susceptibility between strains BJCA002 and BJCA001, we predicted that the two isolates could also differ in other biological features. Next, we examined the morphologies of the two isolates. As shown in **Figure S1**, the cell size of BJCA002 was much smaller than that of BJCA001 in both YCB-BSA and YPD media at 25°C and 37°C.

**Figure 2.**
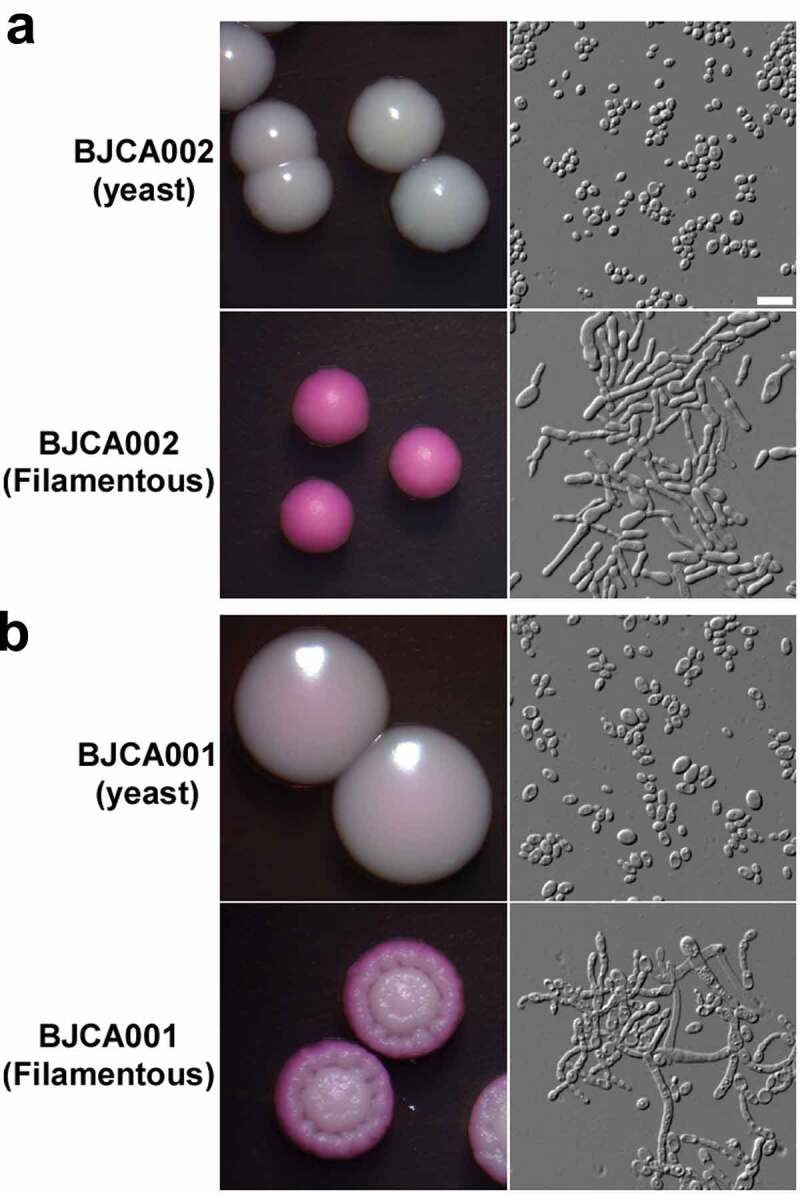
**Colony and cellular morphologies of strains BJCA002** (a) **and BJCA001** (b). Yeast-form or filamentous cells of *C. auris* were plated on YPD medium and incubated at 25°C for 7 days. Scale bar, 10 μm

Filamentous growth is a key feature of virulence [28]. We have previously demonstrated that BJCA001 is able to undergo filamentation [29]. Here we found that this strain was also able to develop filaments on YPD medium at 25°C, although the morphologies of filamentous colonies of BJCA002 and BJCA001 appeared different in the presence of the red dye phloxine B (Figure 2). Similar to BJCA001, the filamentation ability in BJCA002 was heritable. Once the cells became filamentous-competent, this ability was maintained for many generations.

## Strains BJCA002 and BJCA001 differ in the expression of SAPs

**Figure 3.**
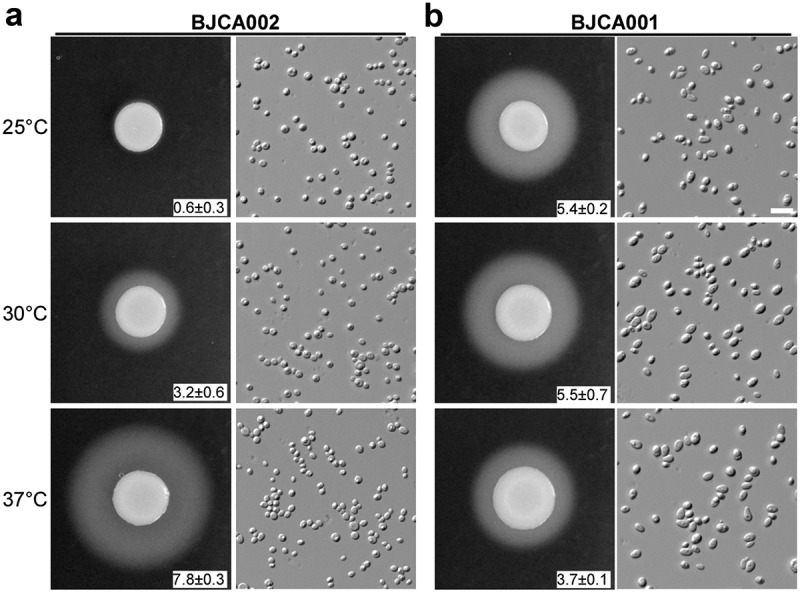
**Comparison of SAP activities of *C. auris* strains BJCA001 and BJCA002**. Approximately 5 × 10^6^ yeast-form cells of BJCA002 (a) or BJCA001 (b) in 5 μL ddH_2_O were spotted onto YCB-BSA medium plates and then incubated at 25°C, 30°C, or 37°C for 7 days. The width of the white precipitation zones (halos) that indicate the level of SAP-mediated BSA hydrolysis activity are shown below the corresponding image (mm). Average values and standard deviations are presented (three repeats). Scale bar, 10 μm

Secreted aspartic proteases (SAPs) are critical for virulence in pathogenic *Candida* species [30]. We next examined SAP expression in the two *C. auris* strains using YCB-BSA medium. As shown in Figure 3, the SAP levels of BJCA001 were comparable at all culture temperatures (25°C, 30°C, and 37°C), whereas the levels of BJCA002 were temperature-dependent. The latter strain secreted higher levels of SAPs at higher temperatures.

## Strain BJCA002 is less virulent than BJCA001 in invertebrate animal and mouse infection models

**Figure 4.**
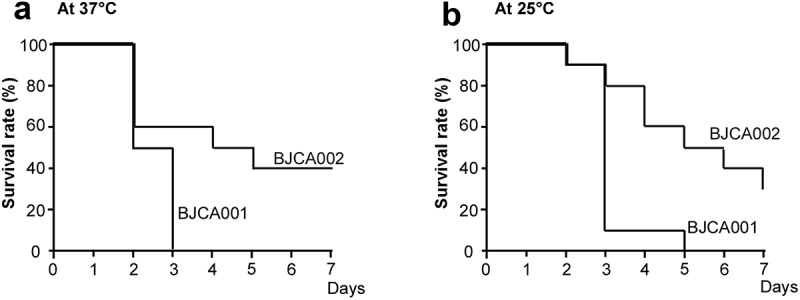
**Virulence of *C. auris* strains BJCA001 and BJCA002 in a *G. mellonella* infection model**. Yeast-form cells of *C. auris* were used. Approximately 1 × 10^6^ *C. auris* cells of each strain were injected into each larva of *G. mellonella*. Ten larvae were injected for each strain. (a) Survival curves at 37°C. (b) Survival curves at 25°C

Since the two *C. auris* strains differed in SAP secretion, we next compared their virulence using two animal models. In the *G. mellonella* invertebrate animal infection model, BJCA002 exhibited less virulence than BJCA001 at both 25°C and 37°C (Figure 4). At 25°C and 37°C, strain BJCA001 (1 x 10^6^ cells/insect) killed all the animals on days 5 and 3 post-infection, respectively. However, at both temperatures, 30% (25°C) or 40% (37°C) of the animals remained viable even at day 7 post-infection with strain BJCA002.

**Figure 5.**
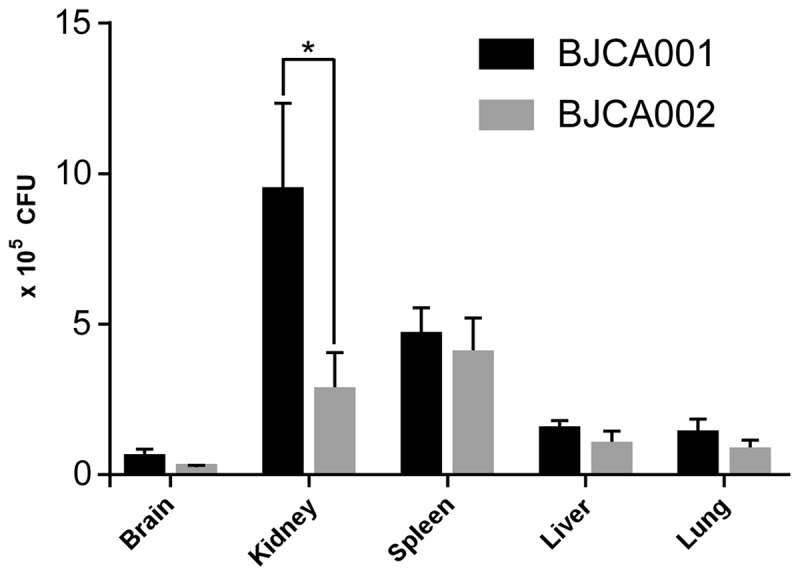
**Virulence of *C. auris* strains BJCA001 and BJCA002 in a mouse systemic infection model**. Fungal burden assays were performed. Five mice were used for infection of each strain. Each mouse was injected with approximately 2 × 10^7^ fungal cells via the tail vein. Mice were killed for CFU assays at 24 h post-infection. *Indicates a significant difference (P value < 0.01, Student’s *t*-test, two-tailed)

To further verify the virulence characteristics, we next performed fungal burden assays using the mouse systemic infection model (Figure 5). The fungal burdens of the two *C. auris* strains were comparable in the brain, spleen, liver, and lung. However, the fungal burden of BJCA002 was significantly lower than that of BJCA001 in the mouse kidney. Taken together, strain BJCA002 was less virulent than BJCA001 in both animal models.

## Comparative analysis of the genomic characteristics of strains BJCA002 and BJCA001

**Figure 6.**
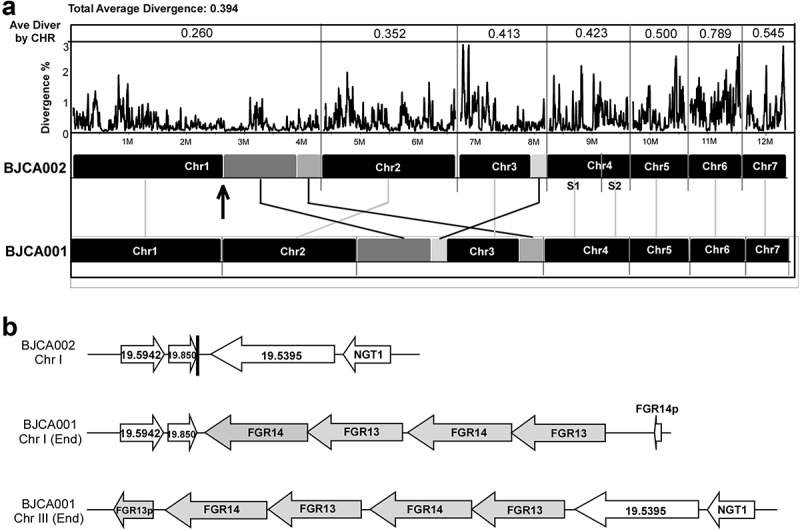
**Whole genome synteny and divergence**. (a) Genome synteny between *C. auris* strains BJCA001 and BJCA002. Black blocks indicate shared syntenic regions of the two genomes, whereas gray blocks indicate large fragment recombination regions. Percentages of divergence for the two genomes and each corresponding pairs of chromosomes are also shown. Every point stands for the SNP density of a 5-kb window and 1-kb step size. (b) Examples of the Zorro3 retrotransposon cluster in strain BJCA001

To reveal the genetic basis of the biological differences between the two *C. auris* strains, we performed whole-genome sequencing assays using next-generation and third-generation sequencing instruments. Highly complete genome assemblies were generated for strains BJCA002 and BJCA001 (Figure 6 and access numbers: CP068451-CP068458 for BJCA001 JAERMV000000000 for BJCA002; https://www.ncbi.nlm.nih.gov/bioproject/PRJNA691446). A summary of the genomic features of the two strains is presented in Table 2. The genome sizes of BJCA002 and BJCA001 were 12.39 Mb and 12.96 Mb, respectively. The genome assemblies were organized in 8 or 7 scaffolds based on a previous study [17]. The divergence values of each corresponding chromosome of the two strains ranged from 0.260 to 0.789, and the average value was 0.394 for the whole genome (Figure 6). As expected, the numbers of genes and introns, GC%, and sizes of intergenic regions were comparable between the two genomes. The SNPs and mutations are presented in supplementary **Dataset S1**.

**Table 2.** Genome assembly information of strains BJCA001 and BJCA002

Strains	BJCA002	BJCA001
Genetic clade	III	I
Total assembly size (Mb)	12.39	12.96
Chromosome/scaffold	8	7
Scaffold N50 (Mb)	2.36	2.39
Scaffold N75 (Mb)	0.94	1.61
GC (%)	45.26	45.11
Number of genes	5397	5517
Intron counts	542	549
Ave. gene size (bp)	1521.7	1553.3
Intergenic ave. (bp)	766.4	788.5
Counts of FGR13 (Zorro3 element)	0.44	46
Counts of FGR14 (Zorro3 element)	3	64
Counts of PF00083 (a general substrate transporter)	38	33
Counts of Endonuclease/exonuclease/phosphatase	10	16

PF: PFAM accession; ORF: CGD Assembly 19/21 Identifier.

The assemblies of the BJCA002 and BJCA001 chromosomes showed a high similarity, and the two genomes were highly syntenic. However, there were some large chromosomal rearrangements (Figure 6). For example, a large chromosomal rearrangement between chromosome 1 of strain BJCA002 and chromosome 3 of BJCA001 involved a translocation of two fragments of 1.26 Mb and 0.41 Mb, respectively. Moreover, a translocation of 139 kb was found in chromosome 3 of the two genomes (Figure 6). This breaking region of strain BJCA001 could have resulted from the insert of a Zorro3 transposon in the chromosome.

## Strains BJCA002 and BJCA001 differ in mating type

*C. auris* is a haploid species. Based on the genomic sequences, we analyzed the mating types of the two strains. BJCA002 was an *MTL*α strain, whereas BJCA001 was an *MTL***a** strain. Consistently, BJCA002 was found to belong to the South African clade, among which all reported isolates carry an *MTL*α locus; strain BJCA001 belongs to the South Asian clade, among which all reported isolates carry an *MTL***a** locus [31].

## Strains BJCA002 and BJCA001 differ in copy numbers of the non-LTR retrotransposon Zorro3

**Figure 7.**
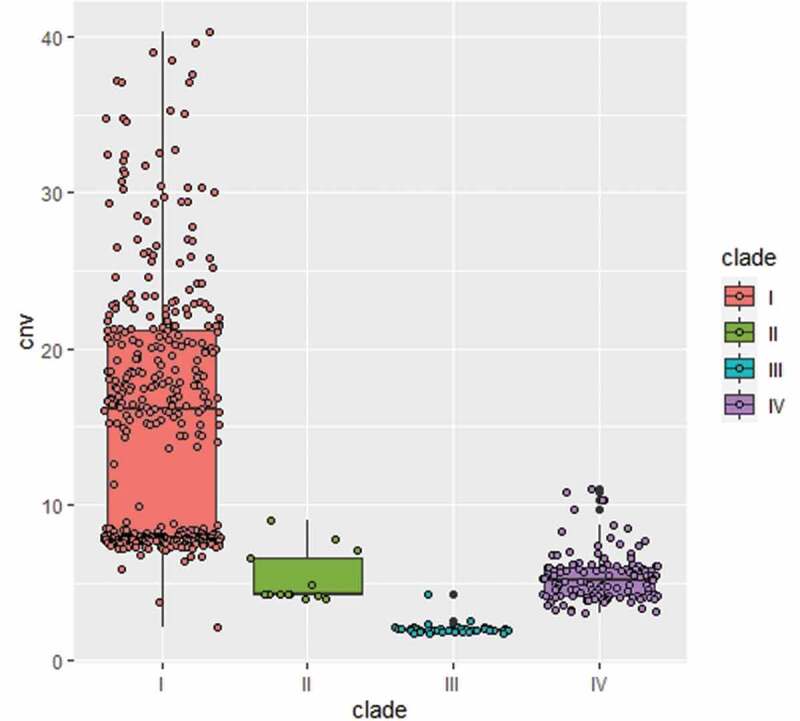
**Variation of the copy numbers of the Zorro3 retrotransposon element *FGR14* in *C. auris* isolates of the four major clades**. Each dot denotes an isolate, and 545 strains were analyzed. The genomic sequences used for analysis were from the NCBI database

A conserved active non-LTR retrotransposon, Zorro3, was identified in *C. auris*. In fungi, the Zorro3 elements often include two genes, *FGR14* and *FGR13. FGR14* encodes a bifunctional enzyme with the activity of reverse transcriptase and endonuclease, whereas *FGR13* encodes a zinc-finger protein with unknown function. Fgr14 has the predicted role in RNA-dependent DNA biosynthetic process, RNA binding, and RNA-directed DNA polymerase activity. Interestingly, we found that the genome of strain BJCA001 contained 64 copies of Zorro3, whereas BJCA002 had only 3 copies (Table 2). The increased genome size of strain BJCA001 could be due to the expansion of ZORRO3 retrotransposon elements. To further confirm the variation in copy numbers among the different clinical strains of *C. auris*, we analyzed 545 genomes available in the NCBI database. As shown in Figure 7, isolates of the South Asian clade (I) generally had higher numbers of Zorro3, whereas isolates of the South African clade (III) had the lowest numbers. The mechanism and function of the expansion of this retrotransposon remains to be investigated.

## Mutations of drug-resistance-associated genes

**Figure 8.**
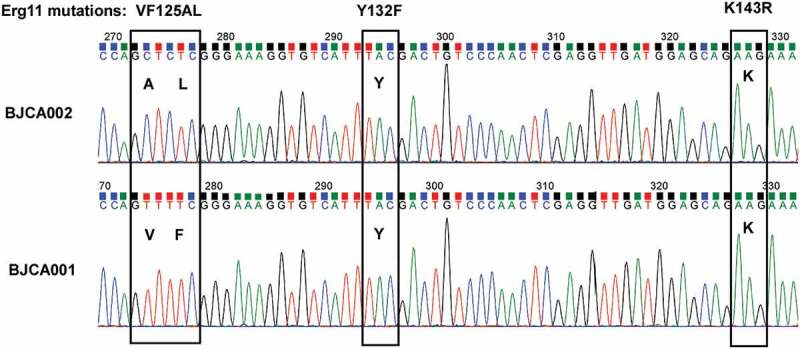
**Analysis of hot-spot mutations in *ERG11.*** Strain BJCA001 carries no mutations in the three reported hot-spot regions, whereas BJCA002 has a VF125AL mutation (V125A and F126L)

SNP analysis revealed several missense mutations in BJCA002 (**Dataset S1**). For example, four missense mutations (G71C, T46S, L279I, and L431W) were found in *CDR4*, which encodes a putative ABC transporter superfamily and could be related to fluconazole resistance in *C. albicans* and *C. auris* [32,33]. Two missense mutations (L286F and V428I) were found in *MDR1* in BJCA002, which encodes an multidrug efflux pump [34]. Mutations were also found in *TAC1* and *MRR1*. Tac1 and Mrr1 could play a role in the transcriptional regulation of *MDR1* and CDR genes. Most importantly, two missense mutations (V125A and F126L) were found in the lanosterol 14-α-demethylase-encoding gene *ERG11* (Figure 8), which could be directly attributed to the fluconazole resistance of strain BJCA002. Taken together, these mutations could provide an explanation for the different antifungal susceptibilities between strains BJCA001 and BJCA002.

## Discussion

We have previously reported the first isolate of *C. auris* (BJCA001) in Beijing, China. Strain BJCA001 was susceptible to all the antifungals tested based on the tentative MIC breakpoints [12]. In the current study, we report a *C. auris* isolate (BJCA002) from the same city that is resistant to fluconazole (MIC = 64 mg/L) and amphotericin B (MIC = 4 mg/L). To explore the diverse characteristics of natural isolates of *C. auris*, we performed a comparative study using the two strains (BJCA001 and BJCA002). We found several distinct biological and genetic features between the two strains, which could contribute to their differences in pathogenesis and antifungal resistance.

Strain BJCA001 belongs to the South Asian clade (I) and contains an *MTL***a** locus, whereas strain BJCA002 belong to the South African clade (III) and contains an *MTL*α locus. *C. auris* isolates of these two distinct lineages have been shown to significantly differ in biological phenotypes and antifungal susceptibility [35]. Here we provide new biological and genetic evidence to reveal the diversity of clinical isolates. For example, strains BJCA001 and BJCA002 differed in cell size and SAP expression, which could be directly associated with virulence. SAPs represent a major virulence factor of pathogenic *Candida* species [30]. Strain BJCA002 expressed a relatively lower level of SAPs at 25°C and 30°C (Figure 3), which might benefit it as a commensal on the skin of the human host. However, both strains were able to develop filaments under certain culture conditions. It remains to be investigated whether this morphological change is also related to pathogenesis in *C. auris* as in other *Candida* species.

To uncover the genetic basis of the difference in antifungal resistance of strains BJCA001 and BJCA002, we sequenced and assembled their genomes (Figure 6 and **Dataset S1**). A couple of chromosome rearrangements were found in the two genomes, which could affect the expression of virulence – and antifungal resistance-associated genes. SNP analysis revealed that the fluconazole-resistant strain BJCA002 carried a number of mutations in potential antifungal resistance-associated genes, including *ERG11, CDR1, MDR1, TAC1*, and *MRR1*. Notably, a mutation of *ERG11* (VF125AL, namely V125A and F126L) in BJCA002 has been previously reported as a hot-spot associated with fluconazole resistance [9,31]. *MDR1* encodes a plasma membrane multidrug efflux pump of the major facilitator superfamily (MFS) that confers resistance to multiple antifungal drugs in *C. albicans*. In clinical isolates of *C. albicans*, a constitutive activation of the *MDR1* gene was often associated with fluconazole resistance and disruption of this gene led to increased susceptibility to fluconazole [36]. Given the close relationship between *C. albicans* and *C. auris*, one would expect that mutations of *MDR1* in this study contribute to the fluconazole resistance of *C. auris*.

We identified an active Zorro3 retrotransposon in the two genomes. A striking difference between the two strains was the presence of 64 copies of the retrotransposon in BJCA001, in contrast to only 3 copies in BJCA002. An analysis of the data available in the NCBI database revealed that *C. auris* isolates of the South Asian clade contain much a greater higher copy number of the Zorro3 retrotransposon, whereas isolates of the South African and the other two clades often have a lower copy number. Some Zorro3 insertions are close to antifungal resistance-associated genes (**Dataset S2**), which may affect the expression of these genes and thus regulate antifungal susceptibility. Interestingly, the transposition of the Zorro2 retrotransposon is activated by miconazole, suggesting that these retrotransposons could be associated with antifungal resistance [37]. In *C. albicans*, the Zorro3 element *FGR14* also regulates filamentous growth [38]. However, how the retrotransposon directly regulates antifungal resistance and morphological changes remains to be investigated. For example, what are the biological effects of the high level expression of the retrotransposon elements *FGR14* and *FGR13*? Does the retrotransposon play a role in the rapid evolution and emergence of this fungal pathogen?

Taken together, our comparative study may not reflect the general differences between isolates of the South Asian and South African clades, but it provides new insights into the diversity of natural strains of *C. auris*. Our study demonstrated the existence of at least two different genetic clades of *C. auris* in Beijing, China. Biological and genomic analyses indicated that they differed in a number of aspects, including the cell phenotype, SAP secretion, virulence, antifungal resistance, and genetic basis. These variations may benefit the survival of the species in distinct ecological niches.

## Supplementary Material

Supplemental Material

## Genomic data

https://www.ncbi.nlm.nih.gov/bioproject/PRJNA691446
