# Supplementary figures and images for: A biological and genomic comparison of a drug-resistant and a drug-susceptible strain of Candida auris isolated from Beijing, China
Source: Virulence. 2021 Jun 1;12(1):1388–99. doi: 10.1080/21505594.2021.1928410 (PMC8172162; doi:10.1080/21505594.2021.1928410)

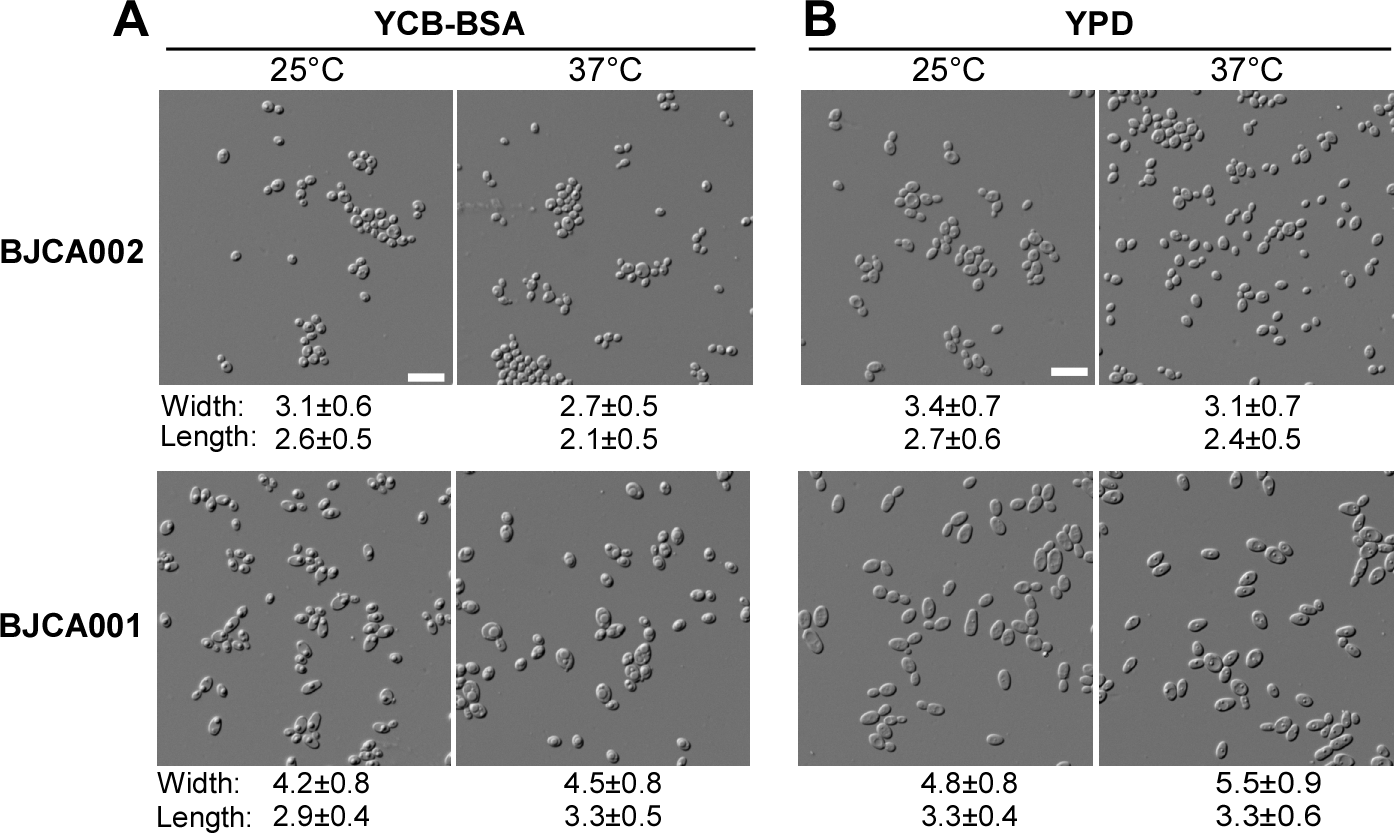

Supplement: Supplemental Material [file KVIR_A_1928410_SM5506.zip › Figure_S1_0105_2021_cell_size.tif]
